# Efficacy of sodium hypochlorite and peracetic acid in reducing cross‐contamination during washing of baby spinach at different water quality levels

**DOI:** 10.1111/1750-3841.17657

**Published:** 2025-01-19

**Authors:** Zhujun Gao, Aprajeeta Jha, Claire L. Hudson, Adam L. Hopper, Faith J. Critzer, Shirley A. Micallef, Donald W. Schaffner, Rohan V. Tikekar

**Affiliations:** ^1^ Department of Nutrition and Food Science University of Maryland College Park Maryland USA; ^2^ Department of Plant Science and Landscape Architecture University of Maryland College Park Maryland USA; ^3^ Department of Food Science and Technology University of Georgia Athens Georgia USA; ^4^ Center for Food Safety and Security Systems University of Maryland College Park Maryland USA; ^5^ Department of Food Science Rutgers University New Brunswick New Jersey USA

**Keywords:** antimicrobial, fresh produce, peracetic acid, sodium hypochlorite

## Abstract

**Abstract:**

We evaluated the antimicrobial performance of sodium hypochlorite (NaOCl) and peracetic acid (PAA) during washing of baby spinach in water of varying levels of organic load, as measured by its chemical oxygen demand (COD). *Escherichia coli* TVS353 was spot inoculated onto one unwashed leaf. Sanitizers were added into water with preadjusted COD (300 or 2500 ppm) to achieve concentrations from 20 to 80 ppm. One inoculated leaf was washed with nine uninoculated leaves in 500 mL water (*n* = 6). Bacterial load on inoculated leaves was lowered by sanitizers in a dose‐dependent manner (*p* < 0.05) and the lowest bacterial survivor levels were observed at 80 ppm with 2.7 ± 1.2 and 5.1 ± 0.5 Log MPN/leaf for PAA and NaOCl, respectively, at low CODs. PAA was more effective in reducing bacterial load from the inoculated leaf than NaOCl at high CODs (*p* < 0.05), with 2.9 ± 2.8 and 5.3 ± 0.8 Log MPN/leaf survivors for PAA and NaOCl, respectively. At 80 ppm sanitizer levels, the bacteria was not detected in wash water at any condition but was detected at 20 and 40 ppm at high CODs. The lowest levels of bacteria transferred to uninoculated leaves were observed at 80 ppm sanitizer, at 0.3 ± 0.2 and 0.2 ± 0.1 Log MPN/leaf for PAA and 1.1 ± 1.0 and 0.3 ± 0.3 Log MPN/leaf for NaOCl at low and high CODs, respectively. The log percentage of bacteria transferred ranged from −1.1 at 0 ppm to over −4.5 at 80 ppm, highlighting a reduction in cross‐contamination by the sanitizers.

**Practical Application:**

This study provides effective data on sanitizer usage to fresh produce industry for ensuring food safety during washing of produce. It evaluated the sanitizer effect in a broad range of scenarios including various sanitizer concentrations, and wash water with low and high organic load that is common when recirculating wash water. The results also revealed the differences in two common sanitizers (PAA and NaOCl) in terms of their effectiveness.

## 1 INTRODUCTION

Baby spinach is one type of fresh produce commonly consumed in the United States. Leafy vegetables (including spinach) are minimally processed and contaminated produce can cause foodborne illnesses (Jaklevic, 2021; Priyanka et al., 2021). *Escherichia coli* is one of the predominant microorganisms often linked to severe outbreaks (Yang & Scharff, 2024). A multistate outbreak caused by *E. coli* O157:H7 linked to baby spinach occurred in November 2021. The outbreak caused 15 illnesses across 10 different states and resulted in four hospitalizations (CDC, 2022). Packaged salad and salad mix are also possible vehicles for the transmission of foodborne pathogens to consumers. Three multistate outbreaks were caused by three distinctly different strains of *E. coli* O157:H7 resulting in a total of 167 illnesses and 85 hospitalizations in 27 states across the country (CDC, 2020) in Fall 2019. Investigation of these outbreaks identified the common strain in each of the outbreaks from fresh‐cut salads containing romaine lettuce and a fecal‐soil composite sample taken from a cattle grate within two miles upslope from the produce farm. Another outbreak in January 2022 was also linked with *E. coli* O157:H7 contamination in packed salad and resulted in 10 illnesses and four hospitalizations in four states (FDA, 2022). All these outbreaks demonstrate the need for improving the antimicrobial efficacy in postharvest washing operations to minimize cross‐contamination risk. Severe *E. coli* outbreaks are often linked with *E. coli* O157:H7; however, surrogates are often used for agricultural studies for safety concerns. *E. coli* TVS353 is an environmental isolate from irrigation water in the Salinas Region, CA, USA (Tomás‐Callejas et al., 2011). It has been used in a number of studies and it has been shown to be a valid surrogate for in‐field experiments (Bardsley et al., 2023, 2022; Kim et al., 2020; Marik et al., 2019; Murphy et al., 2023). It is not pathogenic and is rifampicin‐resistant. Considering the safety concerns and potential scale‐up in‐field studies, we chose this strain.

Sodium hypochlorite (NaOCl) and peracetic acid (C₂H₄O₃; PAA) are commercially available products and widely used in produce washing. Their antimicrobial properties have been well studied (Gil et al., 2019; López‐Gálvez et al., 2020; Singh et al., 2018; Tudela et al., 2019). In peroxyacetic acid‐based sanitizers, the antimicrobial effect was mainly attributed to the oxidative radical species generated by PAA (Kitis, 2004). These reactive oxygen species (ROS) cause bacterial cell damage and inhibit cell recovery (Kitis, 2004). Hypochlorous acid is the primary antimicrobial compound responsible for the inactivation of microorganisms in NaOCl‐based sanitizers, but the hypochlorous acid concentration is influenced by pH, with the hypochlorite ion predominating when the pH is > 7.5. Both hypochlorous acid and the deprotonated hypochlorite ion are considered “free” or available chlorine, but the former has a significantly better antimicrobial capacity. The concentration of available chlorine will be altered by the organic load and other water quality parameters (Chen & Hung, 2017; Gil et al., 2019; Murray et al., 2018).

The quality of produce wash water is of concern as it requires a balance between effective food safety outcomes while meeting regulations, resource availability, and cost. Single‐use water may not be cost‐efficient, but the recirculation and/or reuse of water may lead to higher microbiological risk and organic load build‐up. Researchers have conducted postharvest washing of fresh produce at a wide chemical oxygen demand (COD) range from approximately 100 to 5000 ppm (Fu et al., 2018; Murray et al., 2018; Weng et al., 2016). Washing fresh‐cut produce can lead to an accumulation of organic load, therefore, COD in fresh‐cut produce water tends to be higher compared with whole produce wash water (Murray et al., 2018). An increase in COD can also occur when water is reused to wash multiple batches of produce. This study was designed to assess sanitizer efficacy in water containing varying levels of organic loads, as quantified by COD, as might be expected when washing leafy vegetables. We used 300 and 2500 ppm COD levels to simulate a wide range of postharvest washing conditions.

## 2 MATERIALS AND METHODS

### 2.1 Bacterial strain

The environmental isolate rifampicin‐resistant *E. coli* TVS 353 obtained from Virginia Tech University was used throughout the study. One loopful of *E. coli* TVS 353 was transferred from the frozen (−80°C) culture and grown in 10 mL of tryptic soy broth (Catalog No. BD211825, BD Bacto) with 80 ppm rifampicin (Catalog No. R0079, TCI America, Inc.) (TSBR) at 37°C for 24 h. The culture was then streaked onto tryptic soy agar (Catalog No. BD 236,920, BD Difco) with 80 ppm rifampicin (TSAR) and incubated at 37°C for 24 h. This served as the working culture and was subcultured weekly on TSAR.

### 2.2 Baby spinach leaves

Unwashed baby spinach leaves (cultivars ‘Space’ or ‘Renegade’) were obtained weekly from a local farm and used throughout the study. Initial testing was conducted on spinach to ensure the absence of any rifampicin‐resistant lactose fermenting bacteria. The spinach leaves were stored at 4°C and used within 2 days of harvesting. Each sanitizer treatment was examined by six replicate experiments using the same batch of spinach leaves. Eleven baby spinach leaves, between 5 and 8 cm in length, were selected for each replicate and their total weight was recorded. While average weights changed over the season, leaf surface areas remained consistent.

### 2.3 Sanitizers

Sodium hypochlorite (NaOCl; DECCO US Post—Harvest Inc.) and peroxyacetic acid (PAA; SaniDate 15.0, BioSafe Systems, LLC.) were used throughout the study.

### 2.4 Inoculation of spinach leaves

A single colony from the working culture was picked, inoculated in 10 mL of TSBR, and incubated at 37°C for 20–24 h 2 days prior to experimentation. Two hundred microliters of culture broth were spread plated on TSAR and incubated at 37°C for 20–24 h 1 day prior to experimentation. Bacterial cells were collected by transferring 5 mL of 0.1% peptone water (Catalog No. CM0009, OXOID Ltd.) (PW) onto the culture plate and colonies were scraped off using a sterile L‐shape spreader on the day of the experiment and served as the inoculum. The inoculum was diluted to 10^−1^ using 0.1% PW and the absorbance was measured at 600 nm. The target OD_600_ value range was 1.5−1.8 which was approximately 10 log CFU/mL of bacteria, and the inoculum was adjusted accordingly. The inoculum was then serially diluted and plated on TSAR for enumeration. Six leaves (two leaves from each replicate) were spot inoculated with 20 µL of inoculum onto the center of the adaxial surface to reach 8 Log MPN/leaf. The inoculated leaves were dried in a biosafety cabinet for 1−2 h until the inoculum was visibly dry.

### 2.5 Simulated wash water

Simulated spinach wash water was prepared using the same source of spinach leaves and potting mix (Professional Growing Mix from Sun Gro Horticulture). One hundred grams of baby spinach leaves were boiled in a pot with 600 mL of water for 1–2 min to inactivate bioactive compounds. The leaf‐water mixture was mixed with 25 g of potting mix after boiling and transferred to a juice blender (Vitamix Explorian). The mixture was blended for 1 min and then filtered once through a four‐layer cheesecloth and twice through cotton balls. The filtered wash water stock suspension was autoclaved at 121°C for 15 min and stored at −20°C (for up to 2 months). Approximately 3.5 L of wash water was prepared by diluting the stock with deionized water to reach 300 ± 50 ppm COD level, and a sample from the prepared wash water was taken for COD measurement 1 day prior to the experiment. COD levels were measured using triplicated mercury‐free COD2 Digestion Vials (Product #2565125, HACH), digested in DRB200 Digital Reactor Block (Product #DRB200‐02, HACH), and quantified with a UV‐Vis spectrophotometer (Product #DR5000, HACH) following the manufacture manual (DOC316.53.01099) (HACH, 2021). Wash water was adjusted to 300 ± 50 ppm based on COD results. The prepared wash water was stored at 4°C for the experiment on the next day. The solid‐to‐water ratio was increased to achieve 2500 ppm COD while keeping the leaf‐to‐soil ratio to be 4:1. The stock and wash water preparation procedure was the same as 300 ppm COD water, and the final wash water was diluted 10 times for COD measurement. In each replicate experiment, five hundred and fifty milliliters of prepared wash water was transferred into a beaker and appropriate volumes of sanitizer were added to achieve desired concentrations of each sanitizer‐free chlorine or PAA at 0 (control), 20, 40, and 80 ppm. The pH of each solution was adjusted to 6.5 with phosphoric acid (Catalog No. A242‐500, Fisher Scientific) for chlorine treatments. Oxidation reduction potential (H198121, Hanna Instruments), conductivity (Catalog No. 15078201, Fisher Scientific), turbidity expressed in Nephelometric Turbidity Units (NTU) (TB 250 WL, Tintometer Inc.), temperature (Catalog No. 15078201, Fisher Scientific), pH (AB15, Fisher Scientific), and sanitizer concentrations (Catalog No. 7514‐01, LaMotte; Catalog No. TK7450‐Z, AquaPhoenix) were determined via corresponding instrument and assays before washing. Five hundred milliliters of final wash water with sanitizer was transferred to a square‐shaped glass container with a lid for later use.

### 2.6 Cross‐contamination washing

Five hundred milliliters of wash water in a glass container (17.1 cm L × 17.1 cm W × 7.6 cm H, 946 mL) was placed onto an orbital shaker. Nine uninoculated spinach leaves were first randomly placed into the water. A single marked inoculated leaf was added, adaxial side up into the wash water. The lid was immediately closed, and the orbital shaker immediately started at 100 rpm. One milliliter of neutralizer solution (0.35 g/mL sodium metabisulfite [Catalog No. S244‐500, Fisher Scientific]) was then added to the washing container to neutralize the sanitizer after 1 min of washing, and the shaking was restarted for 10 more seconds to neutralize the sanitizer in the water. The inoculated leaf was collected first with a sanitized tweezer into a stomacher bag containing 10 mL of PW + 0.2% (W/V) tween 80 (Catalog No. T0546500G, TCI America, Inc.). The uninoculated leaves were collected individually and transferred into stomacher bags containing 10 mL of PW + 0.2% tween 80 also using sanitized tweezers. The inoculated unwashed leaf was processed in the same manner. Ten milliliters of used wash water was also dispensed into an empty stomacher bag for the Most Probable Number (MPN) analysis.

### 2.7 Determination of bacterial populations

Leaf samples in stomacher bags were processed with stomaching (Seward Ltd., Stomacher 80 Biomaste) at 230 rpm for 1 min to dislodge bacteria. The leaf wash water samples were then serially diluted with 0.1% PW. The MPN method was used for consistency in recovering the low bacterial levels in some treatments. Proper dilutions of each sample were enumerated with a USDA FSIS MPN method modified to use lactose peptone broth with rifampicin (Gao et al., 2024). All the MPN boxes were incubated at 37°C for 24 h. The MPN boxes were examined based on color change after the 24‐h incubation: brown color was positive, and purple color was negative. The MPN measurement range was 0.3−110 MPN/mL. All the stomacher bags were then stored at 4°C. If the MPN box results fell out of the detection range, the samples were repeated with a lower dilution to obtain the bacterial estimates within 24 h of the experiment.

### 2.8 Data analysis

The collected data were entered into Excel spreadsheets. All data points below the lower detection limit were assigned a value of 0.5 × 0.3 = 0.15 MPN/leaf or MPN/mL = −0.8 Log MPN/leaf or Log MPN/mL and then used for analysis. Percentages of transfer to produce and water were calculated using the formulas below:

- Percentage of transfer to produce
$$\begin{array}{ccc} & & \left(\frac{\mathrm{MPN}/\mathrm{leaf}\;\mathrm{on}\;\mathrm{uninoculated}\;\mathrm{leaf}\;\mathrm{after}\;\mathrm{washing}}{\mathrm{MPN}/\mathrm{leaf}\;\mathrm{on}\;\mathrm{inoculated}\;\mathrm{leaf}\;\mathrm{that}\;\mathrm{was}\;\mathrm{not}\;\mathrm{washed}}\right)\\ & & \;\times \;100\% \end{array}$$
- Percentage of transfer to water
$$\left(\frac{\mathrm{MPN}/\mathrm{mL}\;\mathrm{in}\;\mathrm{water}\;\mathrm{after}\;\mathrm{washing}\times \;\mathrm{total}\;\mathrm{volume}\;\mathrm{of}\;\mathrm{simulated}\;\mathrm{wash}\;\mathrm{water}}{\mathrm{MPN}/\mathrm{leaf}\;\mathrm{on}\;\mathrm{inoculated}\;\mathrm{leaf}\;\mathrm{that}\;\mathrm{was}\;\mathrm{not}\;\mathrm{washed}}\right)\times 100\%$$

The MPN results and the percentages of transfer to produce and water were also transformed into Log_10_ form for log‐reduction data analysis. The bacterial levels were compared with a one‐way analysis of variance. Tukey's honest significant difference post hoc test was used to determine significant differences among treatments at a significance level of *p *< 0.05.

## 3 RESULTS AND DISCUSSION

### 3.1 Water quality and sanitizer application

Inoculated spinach leaves had a starting concentration of 8.10 ± 0.37 Log MPN/leaf over all trials. The actual measured COD water levels were 311.17 ± 16.3 ppm and 2505.76 ± 29.15 ppm, respectively, over all trials. Water quality parameters before washing with PAA and NaOCl at different concentrations are given in Tables 1 and 2, respectively. The parameters with the largest differences between the two COD levels were turbidity and conductivity. Water had significantly higher turbidity and conductivity (*p *< 0.05) at 2500 ppm COD. Oxidation reduction potential and conductivity generally increased as NaOCl levels increased, but similar changes were not observed with PAA at either COD. The addition of PAA reduced the water pH to 3.97 ± 0.03 in 80 ppm at 300 ppm COD. In 2500 ppm COD water, the addition of a large amount of spinach leaf tissues increased the amount of naturally presented acids in the water, and it led to a lower pH level in control group water (4.94 ± 0.09). The pH of the chlorine solution was adjusted to maintain a pH of 6.5 and counteract the addition of NaOCl, as mentioned above.

**TABLE 1:** Water quality parameters for PAA treatments.

		Sanitizer concentration			
Water COD (ppm)	Water quality parameter	0 ppm	20 ppm	40 ppm	80 ppm
300	Oxidation reduction potential (mV)	292.8 ± 12.6A	463.0 ± 9.6B	478.5 ± 10.9B	511.3 ± 2.7C
2500		344.0 ± 12.3A	380.7 ± 1.2B	390.5 ± 3.3B	408.2 ± 5.9C
300	pH	6.62 ± 0.07A	4.58 ± 0.07B	4.41 ± 0.11C	3.97 ± 0.03D
2500		4.94 ± 0.09A	4.92 ± 0.05A	4.67 ± 0.04B	4.48 ± 0.07C
300	Conductivity	298.3 ± 7.7 (µs)ABCD	271.2 ± 11.7 (µs)B	308.5 ± 9.2 (µs)C	291.8 ± 6.4 (µs)D
2500		2.32 ± 0.13 (ms)A	2.67 ± 0.06 (ms)BC	2.65 ± 1.12 (ms)B	2.86 ± 0.18 (ms)C
300	Temperature (°C)	15.00 ± 1.50A	14.25 ± 0.65A	16.10 ± 1.80A	15.65 ± 1.33A
2500		13.35 ± 0.74A	13.77 ± 0.54A	14.35 ± 0.45AB	15.45 ± 1.80B
300	Turbidity (NTU)	39.03 ± 6.57A	50.33 ± 5.28B	39.16 ± 4.51A	58.83 ± 9.00B
2500		479.47 ± 7.83A	391.15 ± 7.48B	393.37 ± 10.15B	524.1 ± 30.34C
300	Measured PAA level (ppm)	N/A	19.5 ± 1.05	39.5 ± 2.81	78.3 ± 4.1
2500		N/A	20.8 ± 2.0	40.8 ± 3.8	80.8 ± 4.9

*Note*: Values are expressed as mean ± standard deviation. Means with different upper‐case letters within the same row are significantly different (*p *< 0.05).

**TABLE 2:** Water quality parameters for NaOCl treatments.

		Sanitizer concentration			
Water COD (ppm)	Water quality parameter	0 ppm	20 ppm	40 ppm	80 ppm
300	Oxidation reduction potential (mV)	292.8 ± 12.6A	852.7 ± 32.7B	894.7 ± 10.6B	641.0 ± 127.0C
2500		344.0 ± 12.3A	643.0 ± 37.2B	880.8 ± 20.4C	890.17 ± 23.7C
300	pH	6.62 ± 0.07A	6.53 ± 0.07B	6.45 ± 0.06B	6.40 ± 0.08B
2500		4.94 ± 0.09A	6.70 ± 0.16B	6.53 ± 0.17B	6.64 ± 0.09B
300	Conductivity	298.3 ± 7.7 (µs)A	1639.5 ± 61.2 (µs)B	1622.5 ± 89.8 (µs)B	2560 ± 21.4 (µs)B
2500		2.32 ± 0.13 (ms)A	13.88 ± 0.95 (ms)B	13.80 ± 1.63 (ms)B	19.10 ± 0.77 (ms)C
300	Temperature (°C)	15.00 ± 1.50A	14.85 ± 1.10A	14.52 ± 1.27A	13.32 ± 1.34A
2500		13.35 ± 0.74A	16.2 ± 0.88B	16.13 ± 0.53B	19.6 ± 0.45C
300	Turbidity (NTU)	39.03 ± 6.57A	52.67 ± 14.53A	44.17 ± 2.87A	41.82 ± 5.35A
2500		479.47 ± 7.83A	539.73 ± 36.92B	496.18 ± 11.04AB	490.2 ± 50.5AB
300	Measured NaOCl level (ppm)	N/A	18.8 ± 1.5	40.8 ± 1.7	81.7 ± 4.1
2500		N/A	20.0 ± 0.0	40.8 ± 0.38	79.2 ± 3.8

*Note*: Values are expressed as mean ± standard deviation. Means with different upper‐case letters within the same row are significantly different (*p *< 0.05).

Water COD level is a critical parameter that affects the antimicrobial efficacy of the sanitizer. COD is defined as the amount of oxygen that is required to completely digest the organic matter in a sample and is most commonly applied to water samples (Najafzadeh & Ghaemi, 2019). High COD levels are generally correlated to higher levels of organic compounds in water, and likely originate from soil particles and from plant tissue juices. Wash water COD levels can vary widely depending on the wash system setup, circulation patterns, and produce variety. We chose 300 ppm to represent a fresh water sample or one with lower organic matter, and 2500 ppm for a water sample representing reused or recirculating water or one with higher organic matter. Previous studies have demonstrated that the antimicrobial effects of PAA and NaOCl can be affected by the organic matter in water, especially the free chlorine released from NaOCl (Ao et al., 2021; Fukuzaki, 2006; Luukkonen & Pehkonen, 2017; Venkobachar et al., 1977; Wang et al., 2019; Zhang et al., 2021). The current study also demonstrated that the presence of organic matter in water interferes with the antimicrobial action of sanitizers. Sanitizers generally showed a higher antimicrobial effect in fresh water (300 ppm COD) than in water with a higher organic load (2500 ppm COD). Lower water COD levels showed a higher efficacy in preventing cross‐contamination to uninoculated leaves (*p *< 0.05) at 40 ppm for both PAA and NaOCl. Our blended spinach leaf technique may have contributed to larger organic loads compared to that arising from soil with high amounts of inorganic sand, for example. The exudates released from plant tissues during the washing process contain a variety of carbohydrates, acids, and other organic compounds, all substantially contributing to the rise in COD (Li et al., 2019; Murray et al., 2018; Zhang et al., 2022). The incremental increase in COD may not be reflected in turbidity and pH measurements, which are the parameters that are commonly monitored in many washing setups and for many commodities (Li et al., 2019). If water COD level build up is primarily due to plant exudates, the measurements of turbidity and pH may not be helpful for water quality determination and ultimately lead to inadequate postharvest treatment in controlling microbial risks.

It is also worth noting that PAA and NaOCl have different mechanisms of action. PAA generates ROS that cause damage in both cell membranes and DNA, oxidize and denature the enzymes and proteins, and interfere with cell recovery pathways (Kitis, 2004; Lin et al., 2024). NaOCl achieves antimicrobial activities mainly by the formation of free chlorine including hypochlorous acid, hydroxide, and hypochlorite ions. The free chlorines are also strongly oxidative species that can cause cell membrane damage, block cellular metabolic pathways, and ultimately cause cell lysis and cell death (Fukuzaki, 2006; Hidalgo et al., 2002; Wang et al., 2019). Both PAA and NaOCl inactivate bacterial cells by their oxidizing mechanisms, while free chlorine from NaOCl shows distinct reactions with organic compounds in wash water (Chen & Hung, 2017). Our experiments required considerably higher amounts of NaOCl solution to achieve the targeted level of free chlorine in 2500 ppm COD wash water than in 300 ppm COD wash water. The required amount of NaOCl solution was roughly dose‐dependent to the water COD levels. To reach 80 ppm free chlorine in water, the actual volume of NaOCl sanitizer added in 2500 ppm COD water was approximately 10 times higher than the volume required in 300 ppm COD water. The required amount of PAA solution varied only by 20% with the increase in water COD level. Our results confirm that substantially lower amounts of PAA versus NaOCl solution are needed to reach target concentrations in high COD water.

### 3.2 Removal and inactivation of bacteria from contaminated spinach

Table 3 compares concentrations of bacterial cells remaining on the inoculated leaves after 1 min of washing in the absence or presence of sanitizers in water with various COD levels. Inoculated cells may dislodge from the leaves with the action of water without sanitizer. When there was no sanitizer in wash water, the inoculated leaves after washing held 6.5 ± 0.3 Log MPN/leaf of bacteria in 300 ppm COD water and 6.8 ± 0.6 Log MPN/leaf in 2500 ppm COD water. This means that 94.5−97.3% of bacterial cells were removed from leaf surfaces during 1 min of washing. There was no statistical difference in bacterial levels between the two COD levels (*p *> 0.05), thus, the organic compounds in water did not change the population of bacterial cells dislodged from leaves when sanitizers were absent. There were fewer *E. coli* detected on inoculated leaves after washing with PAA compared to plain water (*p *< 0.05), with greater reductions occurring with higher PAA concentrations. The same pattern occurred at 2500 ppm COD, although only 80 ppm PAA resulted in significantly lower bacterial populations remaining on the surface of the leaves compared to the control and the 20 and 40 ppm (*p *< 0.05). While PAA showed better efficacy at the lower COD level, this was not the case when NaOCl was used. At 300 ppm COD level, only 80 ppm NaOCl treatment resulted in significantly lower survivors on the leaf compared to control and other concentrations (*p *< 0.05). At either COD level, *E. coli* concentrations remaining on leaves were significantly higher at 80 ppm when NaOCl was used versus PAA (*p *< 0.05). NaOCl treatment at 80 ppm had only a marginally greater effect on bacteria remaining on the leaf surface compared to control and lower concentrations at 300 ppm COD (*p *< 0.05). NaOCl was also significantly less effective than 80 ppm PAA at 2500 COD (*p *< 0.05). These results show that NaOCl was generally less effective in removing or inactivating bacteria from the contaminated produce surface than PAA under the same conditions.

**TABLE 3:** Levels (Log MPN/leaf) of *E. coli* TVS353 found on inoculated leaves after washing.

Sanitizer	COD (ppm)	0 ppm	20 ppm	40 ppm	80 ppm
PAA	300	6.5 ± 0.3*aA*	5.3 ± 1.4*aB*	3.9 ± 3.0*aBC*	2.7 ± 1.2*aC*
	2500	6.8 ± 0.6*aA*	6.0 ± 0.9*aA*	5.8 ± 0.8*bA*	2.9 ± 2.8*aB*
NaOCl	300	6.5 ± 0.3*aA*	5.9 ± 0.7*aA*	6.0 ± 0.6*bA*	5.1 ± 0.5*bB*
	2500	6.8 ± 0.6*aA*	5.7 ± 0.5*aBC*	6.8 ± 1.2*bAB*	5.3 ± 0.8*bC*

*Note*: Values are expressed as mean ± standard deviation. Means with different lower‐case letters within the same column are significantly different (*p *< 0.05). Means with different upper‐case letters within the same row are significantly different (*p *< 0.05). The lower detection limit is −0.52 Log MPN/leaf.

### 3.3 The sanitization of wash water

We quantified *E. coli* TVS353 found in wash water after washing (Table 4). When sanitizer was absent in wash water, more than 5 Log MPN/mL of *E. coli* TVS353 was detected in 500 mL of wash water after 1‐min washing. As the sanitizer levels increased, the bacteria detected in wash water decreased at both COD levels and for both sanitizers. The addition of 20 ppm of either sanitizer was effective in controlling the bacterial levels under 3.3 Log MPN/mL in wash water, and there was no significant difference between the two sanitizers at either COD level. When the sanitizer levels increased to 40 ppm, both PAA and NaOCl yielded log reductions close to or below the lower detection limit (0.3 MPN/mL) in 300 ppm COD water. Forty ppm PAA and NaOCl both showed significantly higher inactivation compared to these of 20 ppm in 2500 ppm COD water (*p *< 0.05) but still yielded viable cells estimated at 1.8 ± 0.6 and 2.4 ± 0.8 Log MPN/mL for PAA and NaOCl in wash water, respectively. Bacteria were not detected at either water COD levels when 80 ppm of either sanitizer was used.

**TABLE 4:** Levels (Log MPN/mL) of *E. coli* TVS353 found in wash water after washing.

Sanitizer	COD (ppm)	0 ppm	20 ppm	40 ppm	80 ppm
PAA	300	5.2 ± 0.6*aA*	2.8 ± 1.4*aB*	−0.8 ± 0*aC*	−0.8 ± 0*aC*
	2500	5.5 ± 0.2*aA*	3.3 ± 0.9*aB*	1.8 ± 0.6*bC*	−0.8 ± 0*aD*
NaOCl	300	5.0 ± 0.6*aA*	2.6 ± 1.8*aB*	−0.6 ± 0.4*aC*	−0.8 ± 0*aC*
	2500	5.5 ± 0.2*aA*	3.3 ± 0.3*aB*	2.4 ± 0.8*bC*	−0.8 ± 0*aD*

*Note*: Values are expressed as mean ± standard deviation. Means with different lower‐case letters within the same column are significantly different (*p *< 0.05). Means with different upper‐case letters within the same row are significantly different (*p *< 0.05). The lower detection limit is −0.52 Log MPN/mL. All data points below the lower detection limit (LDL) were assigned a value of 0.5 × 0.3 = 0.15 MPN/mL = −0.8 Log MPN/mL and then used for analysis.

The choice of sanitizer also changes other water quality parameters for the same initial quality of water. Water turbidity was mainly affected by COD levels in our study. Water pH in all PAA treatments were below 5.0 and the lowest pH level observed was 3.97. The addition of NaOCl drastically increased water pH above 10.0 so water pH had to be adjusted back to 6.5 using phosphoric acid to activate the antimicrobial action of free chlorines. The addition of NaOCl also changes water conductivity where high concentrations of NaOCl increase water conductivity significantly compared to PAA. The impact of these parameters may also influence wastewater treatment options.

### 3.4 The prevention of leaf‐to‐leaf cross‐contamination

We measured the bacterial cross‐contamination from inoculated leaves to uninoculated leaves during the washing process (Table 5). *E. coli* TVS353 was detectable on uninoculated leaves across all sanitizer concentrations at both COD levels regardless of the presence of up to 80 ppm sanitizers. There were 4.8 and 5.3 Log MPN/leaf of bacteria detected on uninoculated leaves in 300 and 2500 ppm COD water without sanitizer, respectively. The addition of 20 ppm PAA or NaOCl significantly reduced bacterial levels detected on uninoculated leaves compared to control water without sanitizer at both 300 and 2500 ppm COD (*p *< 0.05). Twenty ppm PAA and NaOCl showed the same level of antimicrobial effect across the two water types, and *E. coli* TVS353 levels were between 2.5 and 3.2 Log MPN/leaf. When the sanitizer concentration increased to 40 ppm, the lowest bacterial transfer was observed with PAA treatment in 300 ppm COD water. Forty ppm PAA in 2500 ppm COD water showed similar levels of cross‐contamination compared to 40 ppm NaOCl in 300 ppm COD water (*p *> 0.05). These concentrations were significantly lower than those detected in 40 ppm NaOCl and 2500 ppm COD treatment (*p *< 0.05). There was no statistically significant difference in the levels of bacterial transfer across two sanitizers and two water types at the highest sanitizer concentration of 80 ppm (*p *> 0.05). Transferred concentrations of bacteria were all low but remained detectable, and the bacteria concentration in 80 ppm sanitizer treatments were between 0.2 and 1.1 Log MPN/leaf.

**TABLE 5:** Levels (Log MPN/leaf) of *E. coli* TVS353 found on uninoculated leaves after washing.

Sanitizer	COD (ppm)	0 ppm	20 ppm	40 ppm	80 ppm
PAA	300	4.8 ± 0.4*aA*	2.6 ± 1.0*aB*	0.4 ± 0.5*aC*	0.3 ± 0.2*aC*
	2500	5.3 ± 0.2*bA*	3.2 ± 0.8*aB*	2.0 ± 0.9*bC*	0.2 ± 0.1*aD*
NaOCl	300	4.8 ± 0.4*aA*	2.6 ± 1.1*aB*	1.8 ± 1.0*bBC*	1.1 ± 1.0*aC*
	2500	5.3 ± 0.2*bA*	2.5 ± 0.8*aB*	3.1 ± 0.8*cB*	0.3 ± 0.3*aC*

*Note*: Values are expressed as mean ± standard deviation. Means with different lower‐case letters within the same column are significantly different (*p* < 0.05). Means with different upper‐case letters within the same row are significantly different (*p* < 0.05). The lower detection limit is −0.52 Log MPN/leaf.

Commercial sanitizers are widely used in the industry for postharvest produce processing. The primary roles of sanitizers in fresh produce washing are to prevent cross‐contamination of foodborne pathogens and spoilage microorganisms as well as to extend shelf life. The orbital shaker simulated a flume washing system at a lab scale in our study. Published research has shown that both PAA and NaOCl have antimicrobial effects and help prevent cross‐contamination during produce washing (Banach et al., 2015; Chen & Hung, 2017; Gil et al., 2015; López‐Gálvez et al., 2021; Zhang et al., 2022). We focused on comparing the efficacy of two sanitizers at certain concentrations at two disparate water quality scenarios that may be encountered in postharvest processing. Both PAA and NaOCl showed generally improved prevention of cross‐contamination from inoculated leaves to other uninoculated leaves as their concentrations increased. The same effect was also observed in reducing the bacterial levels in wash water. Truchado et al. (2021) conducted a study using 20 ppm NaOCl and 80 ppm PAA washing spinach leaves at around 300 ppm COD level, and our results are comparable to what they observed. NaOCl treatments did not yield as high a reduction of *E. coli* TVS353 from inoculated baby spinach leaves compared to PAA treatments at the same concentration. Based on the results in Table 3, we believe that NaOCl does not effectively dislodge the bacterial cells from the leaf surface, or it cannot directly inactivate the bacterial cells that are attached to leaf surfaces, unlike PAA. We acknowledge that bacterial contamination levels as high as 8 Log CFU/leaf might not be realistic, which is higher than some studies (Allende et al., 2008; Thi‐Van et al., 2019; Tudela et al., 2019), while some other studies were also conducted in the similar inoculum levels (Kilonzo‐Nthenge & Liu, 2019; Niemira, 2007; Singh et al., 2018; Truchado et al., 2021). The sanitizer treatments we studied may be sufficient to eliminate the bacterial risk and keep the target *E. coli* undetectable on noncontaminated leaves when initial contamination levels are low. The current setup can represent a worst‐case scenario, and future studies are required to actually quantify the overall risk at a lower inoculum level. In addition, a small number of uninoculated leaf samples were reanalyzed after overnight refrigeration storage if the initial results were out of range. The storage may cause changes in bacterial levels in the samples, but we assume the impact is minor as they were stored at 4°C in 0.1% PW, which is not an optimal condition for *E. coli* growth.

### 3.5 The changes in percentages of bacterial transfer

**FIGURE 1:**
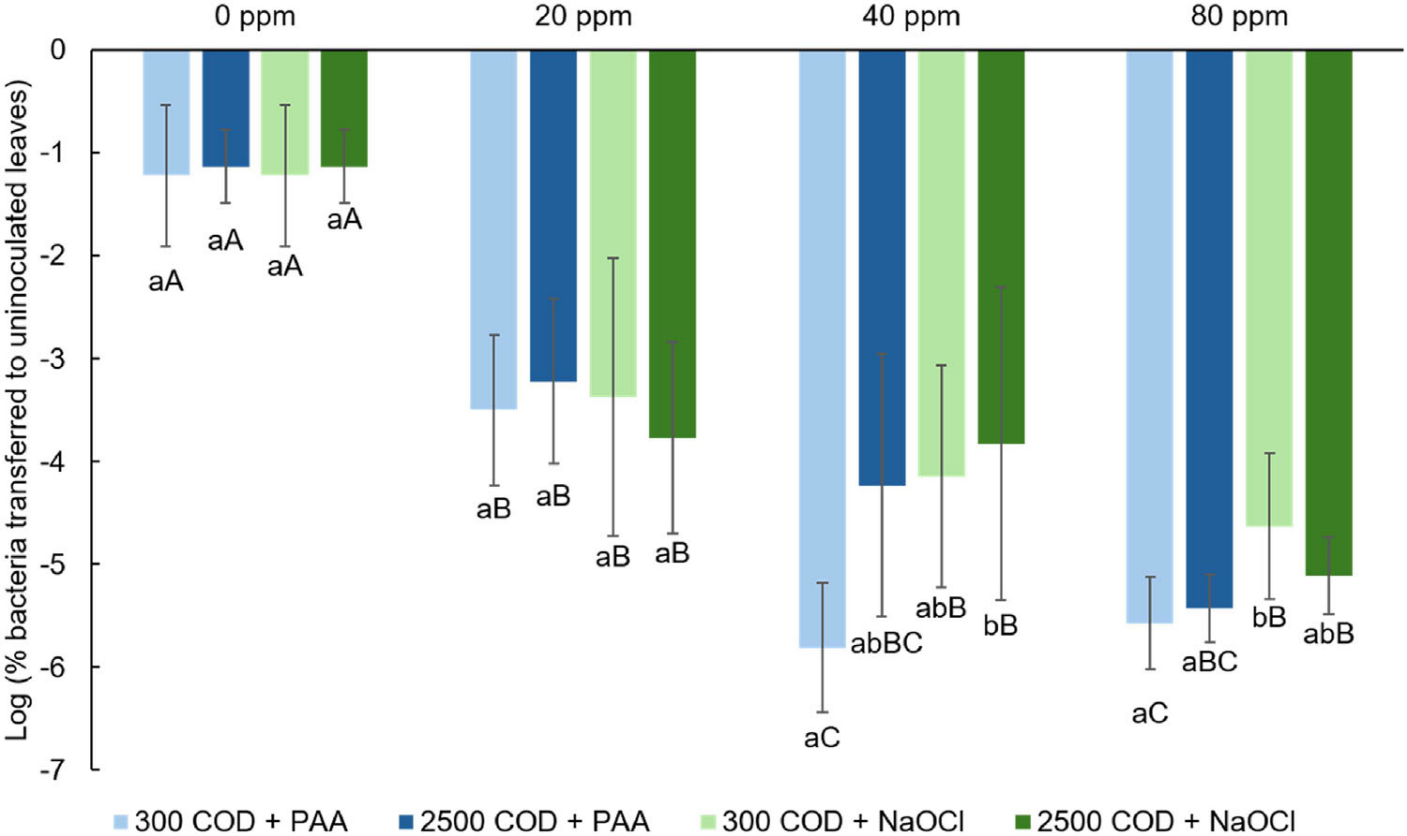
Log percent *E. coli* TVS353 transferred to uninoculated leaves after washing. Values are expressed as mean ± standard deviation. Means with different lower‐case letters within the same sanitizer concentration (same horizontal category) are significantly different (*p* < 0.05). Means with different upper‐case letters within the same water quality and sanitizer combination (same color of bars) are significantly different (*p* < 0.05).

**FIGURE 2:**
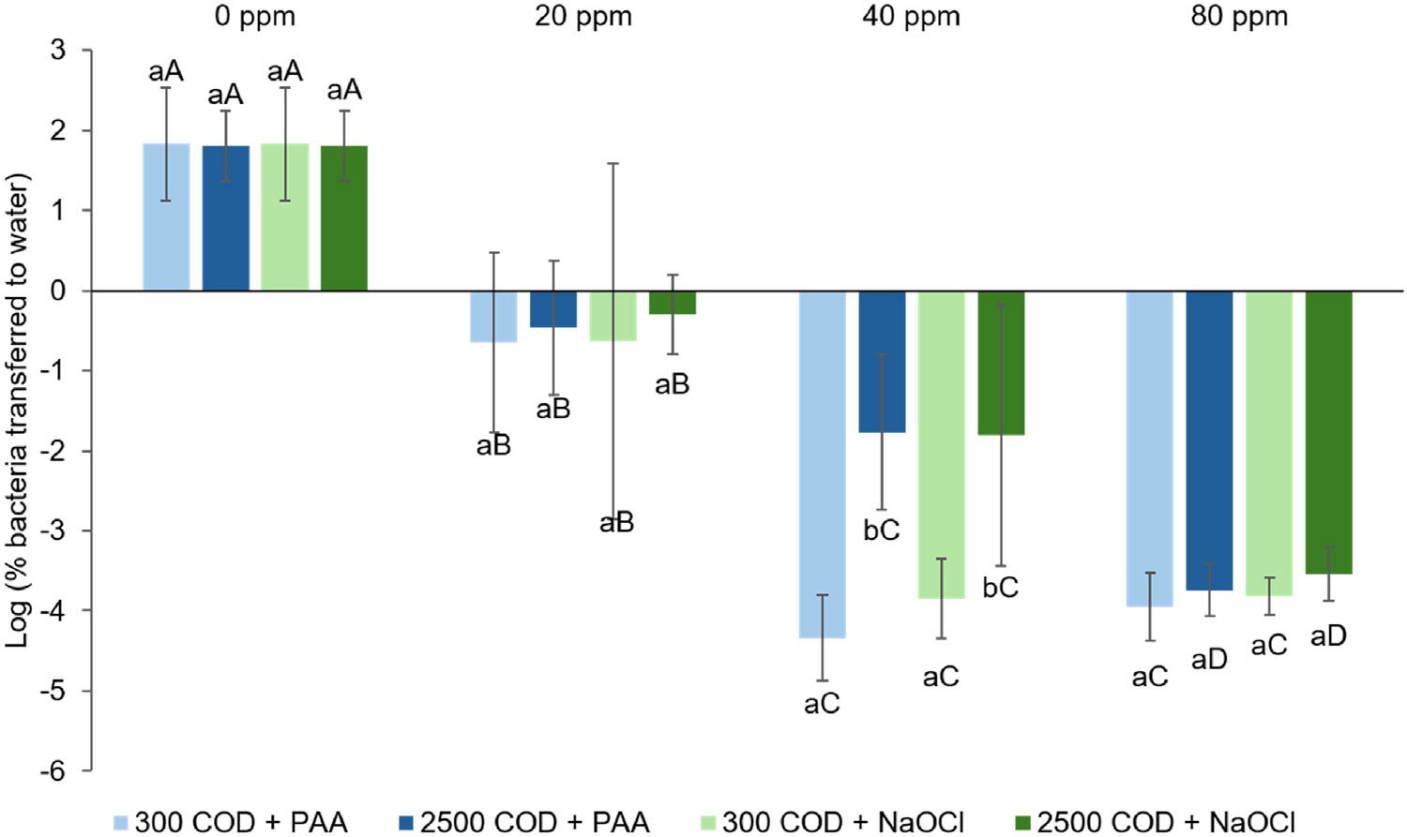
Log percent *E. coli* TVS353 transferred to wash water after washing. Values are expressed as mean ± standard deviation. Means with different lower‐case letters within the same sanitizer concentration (same horizontal category) are significantly different (*p *< 0.05). Means with different upper‐case letters within the same water quality and sanitizer combination (same color of bars) are significantly different (*p *< 0.05).

Lastly, we also calculated the percentages of bacterial transfer to uninoculated leaves (Figure 1) and water (Figure 2) using the original MPN results. The two percentages were converted into Log_10_ numbers to obtain a normal distribution for statistical analysis, and they represent the reduction in bacterial transfer risks. Both percentages of transfer were negatively associated with the sanitizer levels in general. After log transformation, the increase in sanitizer concentration resulted in a significantly higher reduction in bacteria‐water transfer risks (*p *< 0.05), but this trend was not obviously observed in the reduction in bacteria‐leaf transfer risks. The reduction in bacteria‐leaf transfer risks was generally at similar levels when applying the same sanitizer concentrations for both PAA and NaOCl across the two water types (*p *> 0.05), and this trend was also observed in the reduction of bacteria‐water transfer risks.

The lab‐scale flume wash system in this study focused on simulating the water flow and swirl using a smaller volume of water compared to commercial systems. The total washing time in this study was determined to be 1 min. In an industrial flume wash system, the washing time may be longer than 1 min depending on the actual system setup (Bornhorst et al., 2018; Huang et al., 2018). Extended washing times may decrease bacterial concentration in water, on contaminated leaves, and cross‐contamination to uninoculated leaves. Longer washing times may allow lower sanitizer concentrations to achieve equivalent levels of bacterial inactivation. Longer washing times may also lead to a loss of sanitizer activity over time. Future studies on bacterial inactivation at extended exposure time are recommended for the potential of reducing sanitizer concentrations.

## 4 CONCLUSION

Our study compared PAA and NaOCl at different concentrations during the washing of baby spinach for two water quality scenarios. Both PAA and NaOCl showed increased bacterial inactivation in wash water and prevention of cross‐contamination to uninoculated leaves as sanitizer concentration increased. Both PAA and NaOCl at 80 ppm reduced bacterial levels below −0.52 Log MPN/mL in both water quality scenarios (300 and 2500 ppm COD). Bacteria transferred from contaminated leaves to uncontaminated leaves were below 0.3 Log MPN/leaf for 80 ppm PAA and below 1.1 Log MPN/leaf for 80 ppm NaOCl at both CODs. Eighty ppm PAA had a significantly greater effect on reducing the bacterial concentration on inoculated leaves compared to 80 ppm NaOCl regardless of water COD. Higher COD levels generally reduced sanitizer effectiveness.

## AUTHOR CONTRIBUTIONS

**Zhujun Gao**: Conceptualization; methodology; software; data curation; investigation; validation; formal analysis; visualization; writing—original draft; writing—review and editing. **Aprajeeta Jha**: Conceptualization; investigation; writing—review and editing. **Claire L. Hudson**: Investigation; writing—review and editing. **Adam L. Hopper**: Investigation; writing—review and editing. **Faith J. Critzer**: Conceptualization; methodology; writing—review and editing. **Shirley A. Micallef**: Conceptualization; methodology; project administration; writing—review and editing. **Donald W. Schaffner**: Conceptualization; writing—review and editing; data curation; formal analysis. **Rohan V. Tikekar**: Conceptualization; methodology; funding acquisition; resources; project administration; writing—review and editing; supervision.

## CONFLICT OF INTEREST STATEMENT

The authors declare no conflict of interest.

## Data Availability

Data will be made available upon reasonable request.
